# Multi-Locus Sequence Typing of *Bartonella henselae* Isolates from Three Continents Reveals Hypervirulent and Feline-Associated Clones

**DOI:** 10.1371/journal.pone.0001346

**Published:** 2007-12-19

**Authors:** Mardjan Arvand, Edward J. Feil, Michael Giladi, Henri-Jean Boulouis, Juliane Viezens

**Affiliations:** 1 Institut für Medizinische Mikrobiologie, Virologie und Hygiene, Universität Rostock, Rostock, Germany; 2 Department of Biology and Biochemistry, University of Bath, Claverton Down, Bath, United Kingdom; 3 Bernard Pridan Laboratory for Molecular Biology of Infectious Diseases, Tel Aviv Medical Center, Tel Aviv, Israel; 4 Unité Mixte de Recherche, Ecole Nationale Veterinaire d'Alfort, Maisons-Alfort, France; University of British Columbia, Canada

## Abstract

*Bartonella henselae* is a zoonotic pathogen and the causative agent of cat scratch disease and a variety of other disease manifestations in humans. Previous investigations have suggested that a limited subset of *B. henselae* isolates may be associated with human disease. In the present study, 182 human and feline *B. henselae* isolates from Europe, North America and Australia were analysed by multi-locus sequence typing (MLST) to detect any associations between sequence type (ST), host species and geographical distribution of the isolates. A total of 14 sequence types were detected, but over 66% (16/24) of the isolates recovered from human disease corresponded to a single genotype, ST1, and this type was detected in all three continents. In contrast, 27.2% (43/158) of the feline isolates corresponded to ST7, but this ST was not recovered from humans and was restricted to Europe. The difference in host association of STs 1 (human) and 7 (feline) was statistically significant (P≤0.001). eBURST analysis assigned the 14 STs to three clonal lineages, which contained two or more STs, and a singleton comprising ST7. These groups were broadly consistent with a neighbour-joining tree, although splits decomposition analysis was indicative of a history of recombination. These data indicate that *B. henselae* lineages differ in their virulence properties for humans and contribute to a better understanding of the population structure of *B. henselae*.

## Introduction

*Bartonella henselae* is a fastidious bacterium associated with a broad spectrum of clinical disease manifestations in humans, including cat scratch disease (CSD) and bacillary angiomatosis (BA). CSD is characterized by subacute regional lymphadenopathy that usually occurs in immunocompetent individuals [1]. BA is a vasculoproliferative disorder which is predominantly encompassed in immunocompromised patients and often associated with chronic or relapsing bacteremia [2]. Cats represent the natural host and main reservoir for *B. henselae*. Infected animals develop relapsing bacteremia of several months duration without overt clinical symptoms [3].

Isolation of *B. henselae* is hampered by the fastidious nature of the organism. The sensitivity of cultural detection of *B. henselae* from tissues other than blood (e.g. lymph node biopsy specimen) is relatively low. The diagnosis of CSD and most other disease manifestations relies on detection of bacterial DNA in tissue specimens by PCR or serology [4], [5], [6]. Therefore, only few human-derived *B. henselae* isolates are available worldwide [7], [8], [9], [10]. In contrast, Bartonellae can be more easily isolated from the blood of infected cats. Several feline isolates have been collected during prevalence studies from different geographical regions [11], [12], [13], [14], [15]. Thus, feline isolates usually outnumber the human-derived isolates in investigations of the molecular epidemiology of *B. henselae*.

Previous studies have shown a considerable genetic heterogeneity among *B. henselae* isolates by using different DNA fingerprinting methods [10], [16], [17], [18]. The first suggestion that human-associated isolates represent a limited subset of the total *B. henselae* population came from a Dutch study of lymph nodes obtained from CSD patients, which revealed a higher prevalence of isolates displaying the 16S RNA-type I in tissue samples of CSD patients than among feline isolates obtained from the same geographic region [19]. Subsequent studies from Germany [4], [16], [20] and Australia [9] further supported the hypothesis that isolates responsible for human disease are not drawn randomly from the feline reservoir. Recent studies have shown that the delineation of *B. henselae* isolates into two genotypes based on the 16S rRNA sequence is not congruent with phylogenetic classifications using other genetic loci such as *gro*EL, *fts*Z and *rpo*B [18], [21]. In 2003, Iredell et al. developed a MLST scheme for *B. henselae* based on comparison of the nucleotide sequences of nine genetic loci [22]. Analysis of 37 feline and human *B. henselae* isolates from Australia by MLST revealed a considerable genetic diversity among feline isolates, while human isolates were more homogeneous [22].

We have recently validated the use of MLST for the definition of *B. henselae* strains by comparison with pulsed-field gel electrophoresis (PFGE) analysis [23]. MLST is a pangenomic approach that identifies very closely related bacterial isolates and allows the reconstruction of micro-evolutionary events [24]. In the present study, MLST was applied to a larger collection of feline and human *B. henselae* isolates from Europe, Australia and the USA in order to further investigate the association between ST, host species and geographical distribution. The clonal and phylogenetic relationships among the isolates was analysed using three different procedures: i) eBURST was used to define clonal lineages and reconstruct very recent events within each lineage [25], ii) a neighbour-joining tree was reconstructed based on concatenated MLST alleles, and iii) splits decomposition was used to detect inconsistent phylogenetic signals in the data indicative of recombination [26].

## Results

### Assignment of the *B. henselae* isolates to STs

From the 184 isolates studied, 182 isolates were assigned to 14 different STs. Two isolates could not be assigned to a ST because they contained different 16S RNA alleles; they will be described elsewhere. A new allele for *rpoB* was obtained from a feline isolate from Israel and designated as *rpoB*-allele 4. The nucleotide sequence of this allele has been deposited in Genbank (Accession No. EU289215) and the MLST web site (in preparation). Six STs were encountered for the first time in this study and designated as ST9 to ST14 in order of detection. The allelic profile, frequency of isolation and geographical distribution of STs, as well as a reference strain for each ST have been presented in Table 1. The average sequence divergence between all pairwise allelic comparisons was 0.5, 0.6, 0.5, 0.4, 0.3, 0.2, 1 and 0.3 percent for the *rrs*, *bat*R, *fts*Z, *glt*A, *gro*EL, *nlp*D, *rib*C, and *rpo*B locus, respectively.

**Table 1:** Allelic profile, frequency of isolation, and geographic distribution of STs.

ST	Reference strain	Host a	Country of origin b	*rrs*	*bat*R	*glt*A	*fts*Z	*gro*EL	*nlp*D	*rib*C	*rpo*B	Frequency (n)	Distribution c
1	Houston-1	H	USA	1	1	1	1	1	1	1	1	43	AM, AU, EU
2	JR2	H	AU	1	1	1	1	2	1	1	1	1	AU
3	HC62	F	AU	1	2	1	1	2	1	1	1	1	AU
4	HC35	F	AU	2	2	1	1	2	1	1	1	11	AU, EU
5	CA-1	H	USA	2	1	1	1	2	1	1	1	38	AM, AU, EU
6	Urlly8 (Marseille)	H	FR	2	3	2	2	2	1	1	2	28	AM, AU, EU
7	Berlin-2	F	GE	2	4	2	3	1	2	2	1	43	EU
8	I107	F	FR	1	2	1	1	1	1	1	1	7	EU
9	Ber-K143	F	GE	2	1	1	1	1	1	1	1	4	AM, EU
10	G449	F	UK	2	3	2	1	2	1	1	2	2	EU
11	I112	F	FR	2	3	2	2	1	1	1	1	1	EU
12	Is-959	F	IS	2	1	1	1	2	1	1	4	1	EU
13	C27	F	CZ	2	3	1	2	1	1	1	1	1	EU
14	FR96/BK36	F	GE	1	2	1	2	2	1	1	1	1	EU

Reference strain represents the first isolate described or obtained with the allelic profile of a distinct ST.

a F, feline; H, human

b USA, United States of America; AU, Australia; FR, France; GE, Germany; UK, United Kingdom; IS, Israel; CZ, Czech Republic

c Geographical distribution; EU, Europe (including Israel); AM, America; AU, Australia

The Urlly8 (Marseille) isolate displayed the *rpo*B-allele 2 and was assigned to ST6 in the present study (Table 1). These results are in accordance with data by Renesto et al. [27], Iredell et al. [22], and our previous results [23], but differ from the data presented by Lindroos et al. [28], who found an extra single nucleotide polymorphism in the *rpo*B allele of the Urlly8 isolate and assigned it to a new ST. The CA-1 isolate displayed the *rrs*- and *gro*EL-alleles 2 in our study, corresponding to ST5. This is in accordance with previous results of different groups [18], [21], [29], but again differs from the data presented by Lindroos et al. [28], who obtained the *rrs*- and *gro*EL-alleles 1 for the CA-1 isolate and assigned it to ST1.

### Geographical distribution and frequency of STs

**Figure 1:**
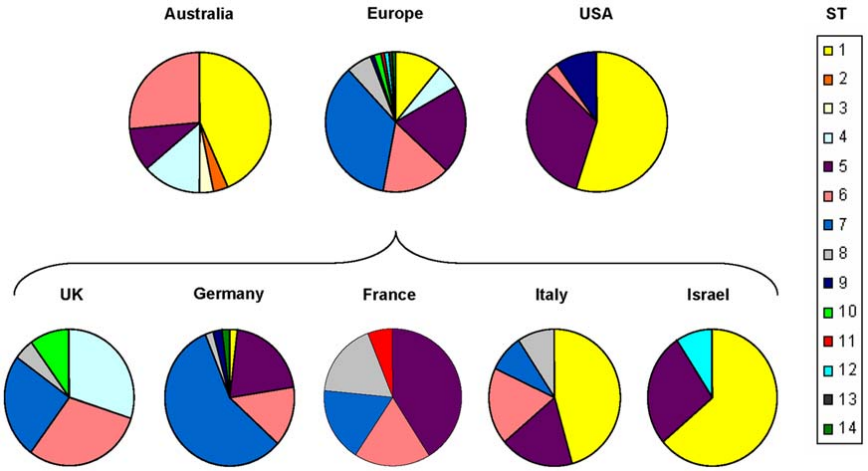
Geographical distribution of *B. henselae* STs in different continents. The lower panel shows the ST distribution in European countries that were represented by at least 10 isolates.

ST1, ST5, ST6, and ST7 were the most common STs, representing 23.6%, 20.9%, 15.4%, and 23.6% of the isolates, respectively. ST1, ST5 and ST6 were isolated in Europe, America and Australia, while ST7 was only distributed in Europe. ST4 and ST9 were distributed in two continents only, being absent from the USA and Australian samples respectively. The less common STs, noted in 1-7 isolates, were found in one continent only. Figure 1 shows the distribution of STs in different continents and among European countries that were represented by more than 10 isolates. The distribution of the major STs in different continents was found to be significantly non-random by chi-square test for STs 1 and 7 (p<0.00001 each), and ST6 (p = 0.039). In contrast, ST5 was evenly distributed among the three continents (p>0.1). The distribution of STs varied also considerably between different countries within Europe. The dominance of ST1 in Italy and Israel, but near absence in France, UK and Germany was particularly striking. To examine this further, we divided the European isolates in two subgroups: i) Mediterranean isolates including all isolates from Israel, Italy, and the Urlly8 (Marseille) isolate, and ii) North-western European isolates (NW-Europe) including all other European isolates. The relative frequency of major STs in different geographic regions was again evaluated by chi-square test (Table 2). This revealed a highly non-random distribution for STs 1 and 7 in Europe, these being over-represented in the Mediterranean and NW Europe, respectively.

**Table 2:** Correlation between ST and geographic origin among 182 *B. henselae* isolates analysed.

ST	Australia	North America	NW-Europe a	Mediterranean b	*P* c
1	13	17	1	12	<0.00001
2	1	0	0	0	0.165 d
3	1	0	0	0	0.165 d
4	4	0	7	0	0.090 d
5	3	10	20	5	0.203
6	8	1	16	3	0.085
7	0	0	42	1	<0.00001
8	0	0	6	1	0.281 d
9	0	3	1	0	0.020 d
10	0	0	2	0	0.630 d
11	0	0	1	0	0.835 d
12	0	0	0	1	0.073 d
13	0	0	1	0	0.835 d
14	0	0	1	0	0.835 d
Total	30	31	98	23	

a North-western Europe including Denmark, Sweden, UK, the Netherlands, Germany, Czech Republic, France (isolates from Paris and Strasbourg), and Switzerland

b Mediterranean region including Italy, Marseille (Urlly8 isolate), and Israel

c As determined by chi square test

d p values have a relative low reliability because of the small number of isolates in this ST

### Relationship between ST and host species

**Figure 2:**
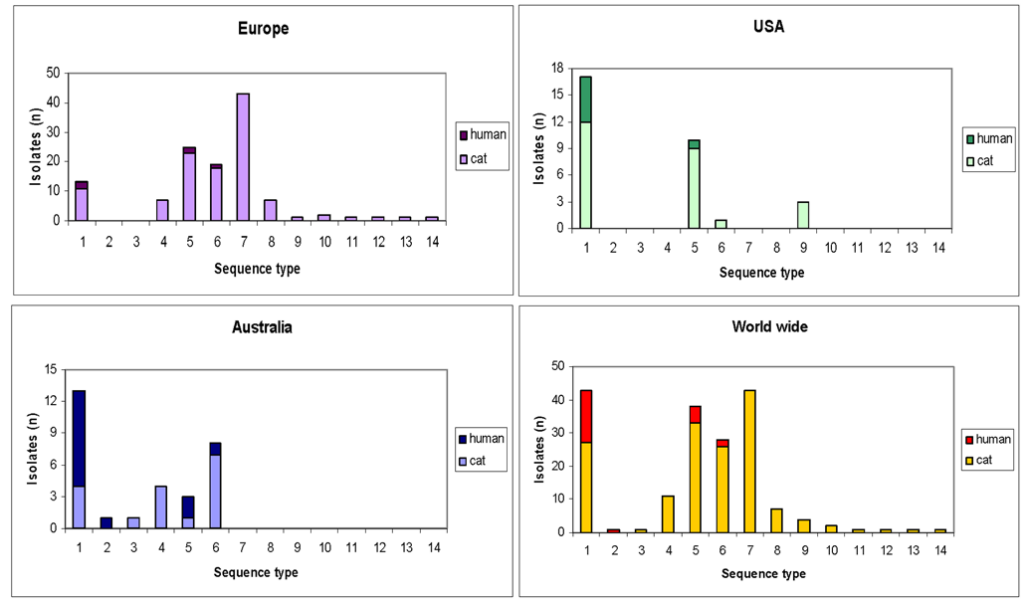
Frequency of feline and human *B. henselae* isolates within each ST in correlation with the geographic origin of the isolates.

The feline isolates (n = 158) were assigned to 13 STs. The human-derived isolates were assigned to 4 STs, including ST1 (16 isolates), ST5 (5 isolates), ST6 (2 isolates), and ST2 (1 isolate). Interestingly, ST7 was not encountered among the human isolates, although it was displayed by 43/121 (35.5%) of the feline isolates from Europe. Figure 2 shows the frequency of feline and human isolates within each ST, along with geographical source. The relative frequency of feline and human isolates within each ST was compared with their frequency in the whole panel by using Fisher's exact test. Human-derived isolates were over proportionally allocated to ST1 and under proportionally associated with ST7 (p≤0.001). The other major STs did not show a disproportional distribution pattern (Table 3).

**Table 3:** Correlation between ST and host species in 182 *B. henselae* isolates analysed.

ST	Feline (%) a	Human (%) b	Total (%) c	*P* d
1	27 (17.1)	16 (66.7)	43 (23.6)	<0.00001
2	0	1 (4.2)	1 (0.5)	0.132
3	1 (0.6)	0	1 (0.5)	1
4	11 (7.0)	0	11 (6)	0.364
5	33 (20.9)	5 (20.8)	38 (20.9)	1
6	26 (16.5)	2 (8.3)	28 (15.4)	0.542
7	43 (27.2)	0	43 (23.6)	0.001
8	7 (4.4)	0	7 (3.9)	0.597
9	4 (2.5)	0	4 (2.2)	1
10	2 (1.3)	0	2 (1.1)	1
11	1 (0.6)	0	1 (0.5)	1
12	1 (0.6)	0	1 (0.5)	1
13	1 (0.6)	0	1 (0.5)	1
14	1 (0.6)	0	1 (0.5)	1
Total	158 (100)	24 (100)	182 (100)	

a Frequency of each ST among feline isolates

b Frequency of each ST among human isolates

c Frequency of an individual ST among all isolates

d As determined by Fisher's exact test

### Evaluation of *eno* for the MLST scheme

The original MLST scheme proposed by Iredell et al. [22] contained *eno*, however, no allelic variability was found in the latter study or in subsequent studies [28]. We therefore decided to determine the *eno* sequence in a selected panel of isolates to evaluate its appropriateness for the *B. henselae* MLST scheme. Fifty isolates that represented every ST and corresponded with their frequency of isolation were analysed. We did not find any allelic diversity among these isolates. Since the selected 50 isolates represent the most heterogeneous *B. henselae* strain collection analysed so far, we conclude that this region of the *eno* gene is not an appropriate target for the *B. henselae* MLST scheme.

### Phylogenetic analysis

**Figure 3:**
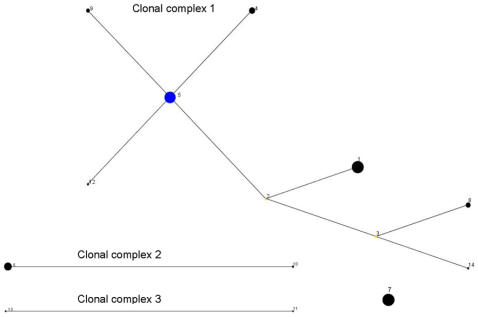
Phylogenetic relationship between different *B. henselae* STs as determined by eBURST. A clonal complex contains STs that have 7 out of 8 alleles in common. ST7 is assigned as a singleton since it differed in 3–7 alleles from all other STs. The size of the circles relates to the frequency of the corresponding ST, and illustrates that the assigned primary founder of the major clonal complex (ST5) is a common clone.

The relationships between the STs was first examined using eBURST (Figure 3), which uses allele profiles rather than sequences and does not attempt to reconstruct the relationships between the different clonal lineages. The majority of the isolates (107/182; 58.8%) corresponded to nine STs, which formed a single clonal complex, Group 1, with ST5 as primary founder. A clonal complex contains STs that have 7 out of 8 alleles in common and a primary founder is the ST with the largest number of single locus variants (SLVs) within a clonal complex. ST5 corresponded to 38/182 (20.8%) of the isolates, which is consistent with its positioning as the founder of STs 9, 4, 12 and 2. The links connecting the other STs in this complex, STs 1, 3, 8 and 14, are less certain, and may have been obscured by recombination. Two doublets were also identified (ST6-ST10, Group 2, and ST13-ST11, Group 3), whilst ST7 differed in two or more genes from every other isolate and was therefore assigned as a singleton.

**Figure 4:**
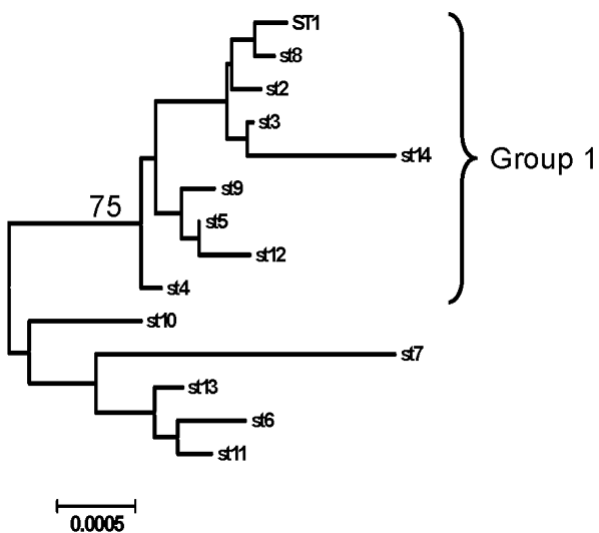
Neighbour-joining tree of the concatenated sequences of *B. henselae* STs as reconstructed by MEGA4. 1,000 bootstrap replicates were used to examine the confidence in the tree. The only node to score above 60 is the one leading to Group 1 (75%, as indicated), indicating that the delineation of Group 1 represents a real division in the *B. henselae* population.

Phylogenetic analysis of the data was carried out by reconstructing a neighbour-joining tree based on concatenated sequences as implemented in MEGA4 [30] (Figure 4). Although the bootstrap support on this tree was generally poor, owing to a paucity of informative sites and possibly a history of recombination, 75% of 1,000 bootstrap trees supported the delineation of Group 1 from the other STs. This is consistent with the hypothesis that it represents a real division within the *B. henselae* population.

**Figure 5:**
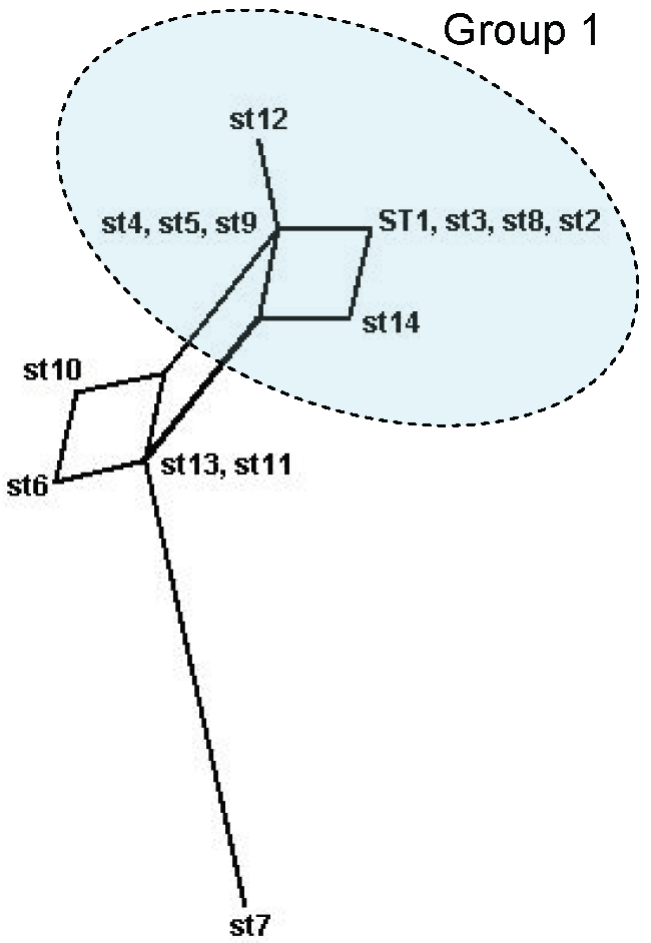
Splits decomposition was used to detect evidence for a past history of recombination in the sequences. The extensive reticulation suggests that recombination has occurred relatively frequently. However, Group 1 remains distinct (as indicated by the filled oval).

Iredell et al [22] noted evidence for recombination from their MLST data, and to explore this issue further we used splits decomposition analysis as implemented in Splitstree4 [26]. The approach examines the degree to which the data correspond to a bifurcating tree, which would indicate limited recombination, or alternatively a network structure, which would be consistent with more frequent recombination. Figure 5 shows that the approach resulted in extensive reticulation between the STs, which is consistent with a history of recombination. Furthermore, the phi test, as implemented in Splitstree4, revealed significant evidence for recombination (P<0.004). Splits decomposition analysis also confirmed the delineation of Group 1, and placed ST7 as a distinct genotype more closely related to Groups 2 and 3 than to Group 1.

## Discussion

In this study, a collection of 182 *B. henselae* isolates from 12 countries and three continents was analysed by MLST to elucidate i) the relationship between ST and host species, ii) the geographical distribution of STs, and iii) the phylogenetic relationship among different STs. To our knowledge, this is the largest *B. henselae* collection that has been analysed by MLST or other molecular typing techniques hitherto. We have tried to minimise sampling artefacts by making every effort to include human isolates from all geographic regions that were represented by feline isolates. However, human isolates were not available from some regions or were outnumbered by feline isolates in other areas. In addition, as the isolates were collected by different investigators in different settings and during a long period of time (approximately 15 years), we can not exclude temporal or seasonal variations or a bias caused by the population (stray versus pet) or breed of the cats examined. Therefore, the panel is still not truly representative of the natural population of *B. henselae*.

Fourteen STs were encountered among 182 *B. henselae* isolates. ST1, ST5, ST6 and ST7 represented major STs and accounted for 83.5% of the isolates. The geographical distribution of STs was not homogeneous. ST1, ST5 and ST6 were found in three continents, suggesting that they may be distributed world wide. In contrast, ST7 was detected only in Europe, suggesting that its distribution may be restricted to Europe. The differences in distribution of STs 1, 6 and 7 on three continents were statistically significant. The distribution of STs varied also between different European countries. ST7 was more prevalent in North and West Europe (UK, Sweden, Denmark, Germany, the Netherlands, France), whereas ST1 was more frequently obtained from the Mediterranean region (Italy, Israel).

We found a significant correlation between distinct STs and human disease. ST1 was statistically significantly associated with human infection, suggesting that it represents a hypervirulent strain. This finding is in accordance with data by Iredell et al. [22], who found a significant association of ST1 with CSD in Australia. They contradict the results by Lindroos et al. [28], who did not find a disproportional association between a distinct ST and human-derived isolates. This discrepancy might be due to differences in size and composition of the panels of isolates. In the latter study, the panel was smaller (n = 38), composed to 60.5% of ST1, and did not contain matched human and feline isolates from the same geographic regions.

ST7 was underrepresented among the human isolates in our study, suggesting that ST7 may be less virulent for humans. However, we can not completely rule out the possibility that the absence of ST7 among the human isolates could be due to a bias in composition of our panel, which contained more feline than human isolates from countries with a higher prevalence of ST7 (e.g. Germany, UK, and France). Further studies with more human-derived isolates from Europe would help to evaluate this hypothesis.

eBURST and phylogenetic analyses were broadly consistent and revealed a major division within the population of *B. henselae*. Of the four predominant genotypes, ST1 and ST5 are related and belong to the major clade, Group 1. ST6 belongs to the minor clade, Group 2, and ST7 is a distinct genotype, probably more closely related to Groups 2 and 3 than to Group 1. Our analysis also supports previous studies which have suggested a history of recombination between the isolates. This is further supported by the “straggly” shape of the major clonal complex as revealed by eBURST. Recent simulation studies have shown that such a structure is indicative of frequent recombination [31].

In summary, our data indicate that different STs of *B. henselae* may vary with regard to virulence for humans. It can be hypothesized that ST1 might possess additional virulence factors, which could encode for a more effective transmission from cats to humans, or a better survival of the pathogen in the human host. It can also be speculated that ST7 may lack one or more virulence determinants, and lower transmission potential may possibly account for the restriction of this genotype to Europe. Future studies using comparative genomic or proteomic approaches could help to identify and characterize these factors. The MLST approach has been previously used for tracking hypervirulent or antibiotic resistant lineages in other bacterial pathogens, e.g. *Neisseria meningitidis*, *Streptococcus pneumoniae* or *Staphylococcus aureus* [24], [25]. MLST data are unambiguous and can be easily transferred electronically between laboratories. Furthermore, MLST can be applied directly to clinical specimens and is therefore not strictly dependent on culture. It can be expected that more MLST data will become available in future, and the establishment of a *B. henselae* site on www.mlst.net should greatly facilitate this.

## Materials and Methods

### Bartonella isolates

One-hundred and eighty four *B. henselae* isolates collected by different investigators in several European countries, Australia, and the USA were analysed. One-hundred and sixty isolates were isolated from feline blood, and 24 isolates were obtained from human tissue specimens, including lymph node, cutaneous BA lesion, and blood. Table 4 summarises the epidemiological data of the isolates studied. Bacteria were stored at −20°C or −80°C until use. The isolates were grown on Columbia blood agar with 5% sheep blood (Becton Dickenson) at 37°C in 5% CO_2_ for 7–14 d, and passaged once on agar prior to isolation of bacterial DNA.

**Table 4:** Geographic origin, host species and clinical source of *B. henselae* isolates.

Country	Cat	Human			Total	Isolate obtained from/reference
		CSD	bacteremia, BA^a^	unknown		
Australia	17	13			30	J. Iredell [22]
Czech Republic	2				2	O. Melter [32]
Denmark	3				3	R. Birtles
France	16	1			17	H-J. Boulouis, P-E. Fournier, Y. Piémont [12], [33]
Germany	49		1		50	our group, A. Sander, D. Schimmel [13], [20], [34]
Israel	9	3			12	M. Giladi
Italy	11				11	M. Fabbi [35]
Netherlands	5				5	A. Bergmans [14]
Sweden	2				2	E. Hjelm
Switzerland	1				1	P-E. Fournier [29]
UK	20				20	R. Birtles [36]
USA	25	1	3	2	31	B. Anderson, L. Guptill [7], [8], [37]
Total	158	18	4		182	

a BA, bacillary angiomatosis

### MLST

Nucleotide sequence data were collected from all *B. henselae* isolates for approximately 320–500 bp fragments of eight genetic loci (16S rRNA [*rrs*], *bat*R, *glt*A, *gro*EL, *fts*Z, *nlp*D, *rib*C, and *rpo*B) as described previously [22], [23]. In addition, the partial sequence of *eno* was determined for 50 isolates [22]. All sequences were determined for both strands and the results were confirmed by repeats when necessary. The reliability of the sequence data was controlled by subjecting 20 randomly selected isolates in a blinded manner as “quality control strains” to MLST analysis. The results of the quality control strains were compared with the data obtained from the “original isolates”. The MLST results were 100% consistent for each pair of quality control strain-original isolate.

### Analysis of MLST Data

The nucleotide sequences were analysed with the DNASTAR Lasergene software package 7 (DNASTAR, Madison, USA). Alleles and STs were assigned in accordance with the published data [22], [23]. New alleles were confirmed by repeats and the sequence was deposited in GenBank (see below). New allelic combinations that were encountered for the first time in this study were assigned to new STs in order of detection.

### Phylogenetic analysis

The definition of clonal complexes and the examination of relationships between STs within clonal complexes were carried out by using eBURST (http://eburst.mlst.net). A neighbour-joining tree was reconstructed from the concatenated MLST alleles using the kimura-2-parameter distance measures as implemented in MEGA4 [30]. Splits decomposition analysis and the phi test were carried out using the default settings in Splitstree4 [26].

### Statistical analysis

Chi square test was used to compare the geographical distribution patterns of major STs. Two-tailed Fisher's exact test was used to compare the frequency of feline and human-derived isolates within a ST with the frequency within the whole panel. *P* values of <0.05 were considered significant.

### Nucleotide sequence accession number

A new *rpo*B allele was encountered from the isolate Is-959 and was designated as *rpo*B-allele 4. This allele contains a single nucleotide variation (G instead of A) at position 711758 of the *B. henselae* Houston-1 chromosome (Accession No. BX897699.1). The *rpo*B-allele 4 sequence has been deposited in GenBank under the Accession No. EU289215. The data were also deposited at http://www.mlst.net (in preparation).
